# Targeting average length of hospital stay as a control measure to decrease COVID-19 hospital-acquired infection in surgical cancer patients

**DOI:** 10.1186/s43046-023-00199-8

**Published:** 2023-11-20

**Authors:** Sarah S. Nasr, Ghada M. Sherif, Maha Abdel Wahab, Hatem Aboelkasem

**Affiliations:** 1 Cancer Epidemiology and Biostatistics Department, National Cancer Institute, Cairo University, Cairo, Egypt; 2 Anesthesia Department, National Cancer Institute, Cairo University, Cairo, Egypt; 3 Surgical Oncology Department, National Cancer Institute, Cairo University, Cairo, Egypt

**Keywords:** Length of hospital stay, COVID-19, Risk assessment, Risk management, Hospital-acquired infection

## Abstract

**Background:**

The global spread of coronaviruses had a great impact on the economic and social situation of most countries. As the backbone of any society, the health sector made a significant contribution through applying emergency risk management plans in order to control the pandemic. Monitoring the average length of hospital stay (ALOS) was an effective way to release the capacity of the health system during this time. The aim was to evaluate the effect of applying risk assessment/management strategies on ALOS and the impact of this ALOS on COVID-19 infection rates among cancer patients.

**Methods:**

This is a prospective cohort study. All admitted cancer patients in 6 surgical departments from January to June 2021 were included.

**Results:**

A total of 1287 patients were admitted to 6 surgical departments during the selected period. About 46% of them had surgery (*n* = 578), while 54% did not have surgery (*n* = 700). Among surgical patients, admission rates were highest in February and head and neck department (24% and 22.1%, respectively), and lowest in April and chest department (12.4% and 8%, respectively). ALOS was significantly different across the 6 months (*p* value < 0.001) with lower ALOS in (April, May, and June) than in (January–February, and March). No significant difference was found across the 6 surgical departments (*p* value = 0.423). Twenty-eight patients became COVID-19 positive after admission, 25 of them (89%) were infected from March to June—during the time of the third wave—and a significant decreasing linear trend (*p* value = 0.009) was found.

**Conclusion:**

ALOS had significantly reduced with commitment to infection control (IC) interventions and recommendations. The significant decreasing trend of COVID-19 infection from March to June (unlike the rising curve of the 3rd COVID-19 wave by that time) could be explained by improvement in ALOS.

## Introduction

Hospital expenditure is the largest contributor to the growth in the national public spending. Given the size of healthcare costs and the constrained government budgets, it is very important that hospitals operate efficiently while still providing high quality care [1].

Acute care addresses health problems that require prompt action including diagnosis and short-term treatment or surgery for severe illnesses and urgent medical conditions. These acute patients should be discharged as soon as they become well enough, while maintaining appropriate standards of care. Acute length of stay (LOS) is the number of days an acute patient spends in the hospital. It is a well-accepted indicator of hospital efficiency and a key driver for the health system’s capacity as it helps keep beds available and reduces the cost per patient [1].

Hospital-acquired infection (HAI) is another important factor affecting patients and society. Risk assessment and management for preventing such infections have become even more critical after the evolution of the COVID-19 pandemic to avoid the enormous burden on the health authorities and the community [2].

Since infection with COVID-19 is more likely to occur in those with lower immunity, underlying medical conditions, or malignant tumors [3], assessment of surgical cancer patients was targeted.

### Study rationale

To the best of our knowledge, literature frequently endorsed COVID-19 infection as a risk factor for prolonged Average length of stay (ALOS) but it lacks references to prior efforts examining the link between ALOS and hospital-acquired COVID-19 infection, especially in malignant tumor patients.

### Study aims/objectives

This study aimed to evaluate the impact of applying IC risk management strategies on the ALOS (January–June 2021) and examine the subsequent effect on COVID-19 infection rates during this period.

### Study hypotheses

- Application and compliance to an appropriate risk management strategy can help in reducing ALOS among cancer patients.
- Minimizing ALOS will reduce hospital-acquired COVID-19 infection among cancer patients.

## Methods

This is a prospective cohort study including 6 surgical departments in the National Cancer Institute (NCI); (Head and Neck, Chest, Gastrointestinal, Orthopedic, Urologic, and Gynecologic departments). The sample size was determined to be inclusive of all admitted patients during the 6-month time period from January to June 2021.

Hospital admission requires a baseline negative COVID-19 PCR swab that should be repeated every 5 days as recommended by the Infection Control (IC) unit for early detection and isolation of positive cases.

Risk assessment was regularly done according to the WHO recommendations [4, 5]. Management of identified risks and monitoring were done across the 6 departments on a daily basis to ensure the best performance and avoid any delay or incompliance as much as possible.

### Risk assessment/management strategies

1. Identify the hazards represented in regular screening to detect positive COVID-19 cases and strict monitoring to detect any violation of applying the IC measures.
2. Decide the vulnerable groups (including health workers, patients, or visitors) and the most common ways of transmission of infection within the hospital facilities.
3. Evaluate the magnitude of infection on a daily basis with appropriate interaction and prompt response.
4. Record the number of positive cases, location, timing, follow-up swab results, and possible source/reason of transmitting the infection.
5. Review risk assessment through a monthly report. This helped to summarize the situation and regularly update the implemented IC precautions that included:
  Implementing standard and transmission-based infection prevention and control precautions (e.g., use the personal protective equipment, environmental cleaning, physical distancing, except when unavoidable during physical examinations and providing care, support for the proper cough etiquette, respiratory hygiene, adequate ventilation, and safe health practice)
  Apply screening protocols for the workforce and patients
  Reduced non-essential surgery
  Restrict visitors and all unneeded activities.
  Promote vaccination of the workforce.

Dates of admission, surgical operation, and first positive PCR swabs (for infected patients after admission) were regularly collected and monthly reports were issued. By the end of June 2021, a collective data set was examined for trends in ALOS and was linked to COVID-19 infection rates.

**Fig. 1:**
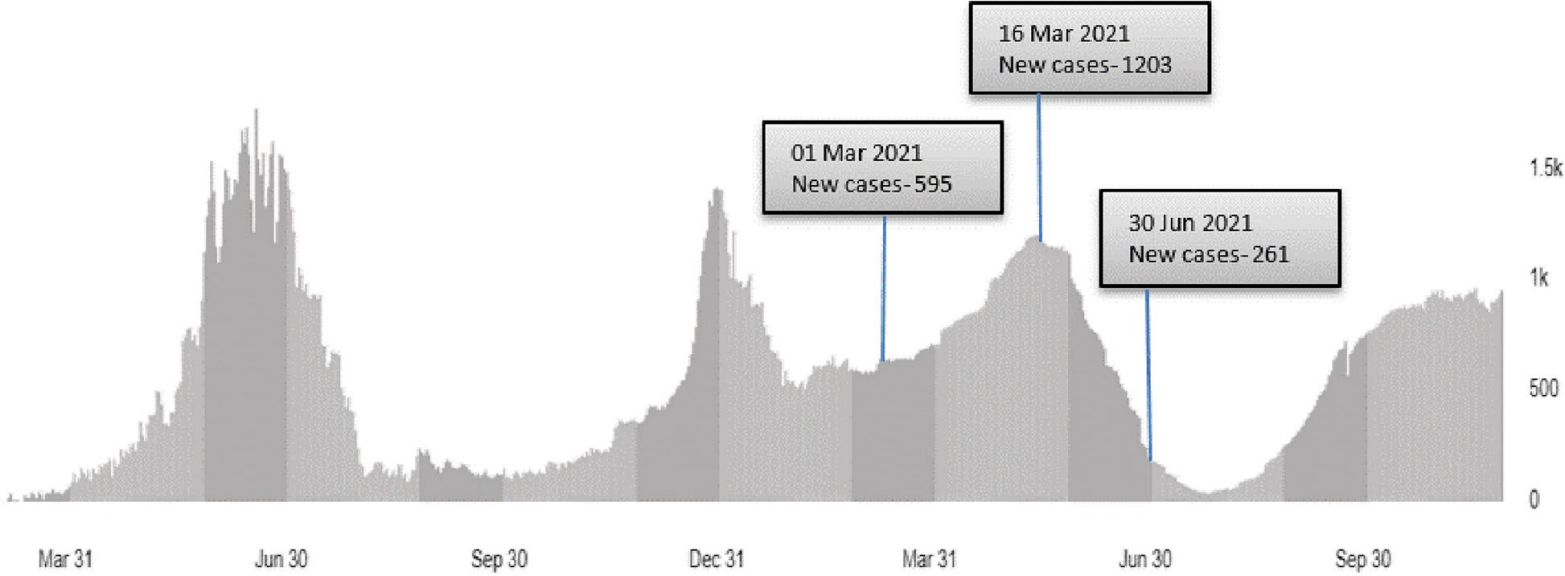
The 3rd wave of COVID-19 infection in Egypt according to WHO statistics

According to the WHO, the 3rd wave of COVID-19 in Egypt began in March 2021, reached a peak in May, and continued till June (Fig. 1), [6]. Subsequently, examination and sub-analysis of infection rates during the wave duration (from March to June) were separately done.

Duration between negative and positive swabs was calculated to assess compliance with the 5-day re-swabbing recommendation.

### Eligibility criteria

All admitted cancer patients in the 6 surgical departments during the selected period were included.

### Statistical methods

Data management and analysis were performed using Statistical Package for Social Sciences (SPSS) vs. 26. Duration of ALOS and negative to positive PCR swabs duration were checked for normality and were statistically described in terms of medians (ranges). COVID-19 state was described as numbers and percentages. ALOS was analyzed in relation to COVID-19 state using the Mann-Whitney *U* test and in relation to months/departments using the Kruskal-Wallis test with Bonferroni adjustment when appropriate. COVID-19 infection rates across the 6 months were analyzed using chi-square and linear by linear association tests. *P* value is always 2-tailed and set significant at < 0.05 level.

## Results

**Fig. 2:**
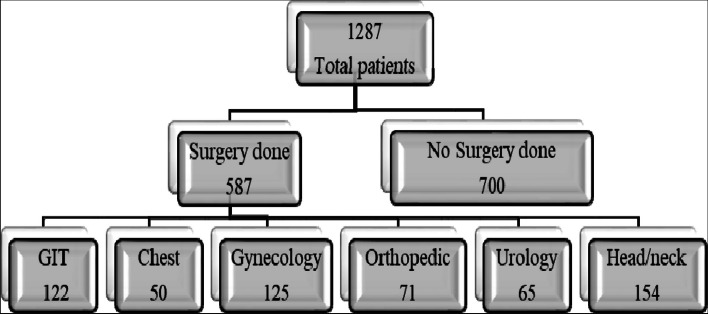
Admitted patients in 6 surgical departments from January to June 2021 (*n* = 1287)

During the period from January to June 1287 patients were admitted. About 46% of them underwent surgery, while the remaining 54% did not have surgery during their admission period (Fig. 2).

Across the 6 months, admission rates were highest in February (22.1%) and lowest in April (12.4%). Among the 6 departments, head and neck department had the highest admission rate (23.9%), while chest department had the lowest admission rate (8.2%).

Analysis of trends in the ALOS and its relation to COVID-19 infection rates showed the following:

### ALOS

#### Preoperative ALOS

For patients who underwent surgery while being admitted (*n* = 587), the pooled preoperative ALOS over all included months and departments was 2 and ranged from 0 to 42 days.

**Fig. 3:**
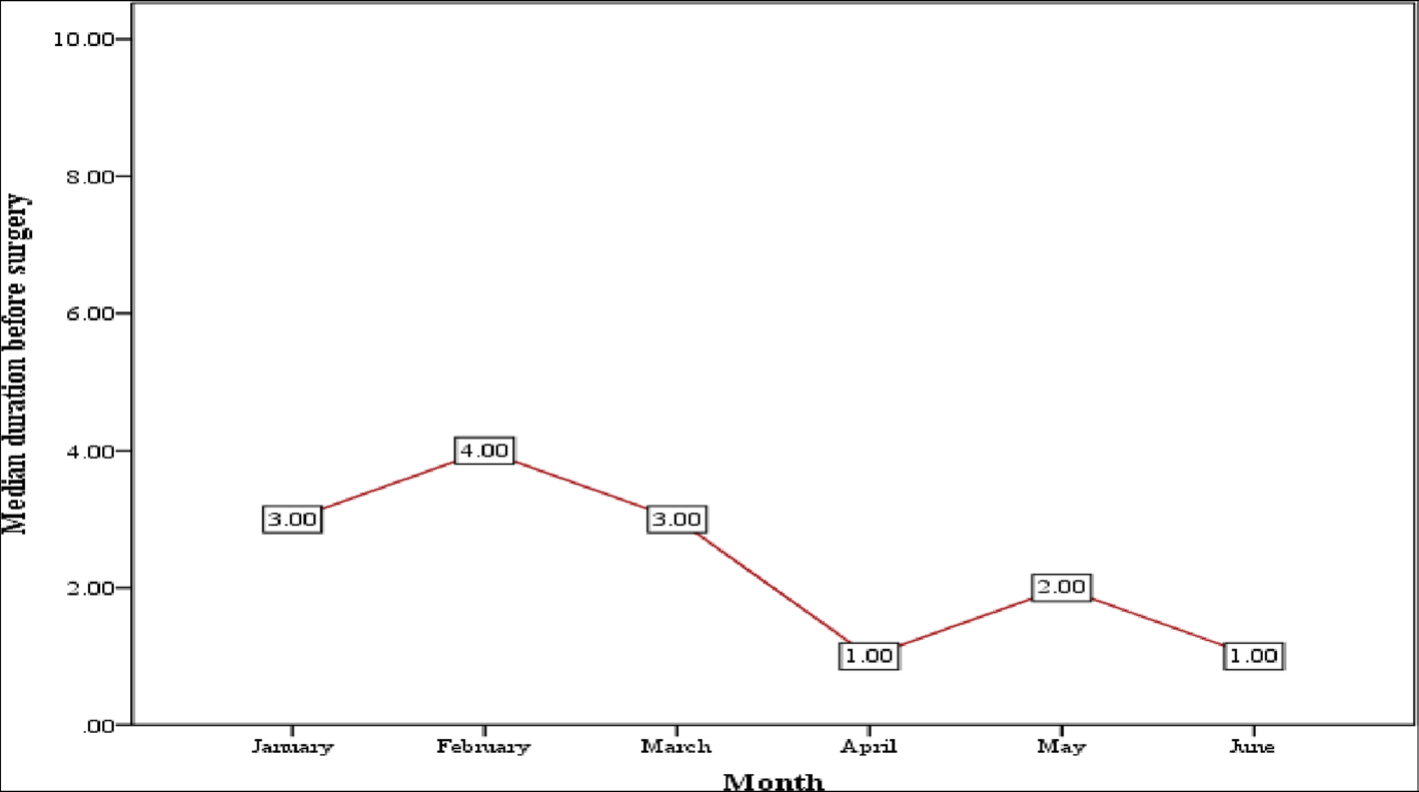
ALOS before surgery per month at NCI during the period from January to June 2021 (*n* = 587)

Table 1 shows a statistically significant decrease in the average preoperative ALOS from January to June, *p* value < 0.001 (Fig. 3). On pairwise Bonferroni adjusted comparison, a significantly lower ALOS was found in June as compared to January (*p* value = 0.014), February (*p* value < 0.001), and March (*p* value = 0.011). May also showed a significantly lower ALOS as compared to February (*p* value < 0.001), while April had lower ALOS as compared to January (*p* value = 0.006), February (*p* value < 0.001), and March (*p* value = 0.005). No statistically significant difference was found between the surgical departments regarding the preoperative ALOS from January to June.

**Table 1:** ALOS before surgery per month at NCI during the period from January to June 2021 (*n* = 587)

	LOS before surgery		
	Median	(Range)	*p* value^a^
Month			< 0.001^*^
January	3.0	(0.0–17.0)	
February	4.0	(0.0–32.0)	
March	3.0	(0.0–42.0)	
April	1.0	(0.0–35.0)	
May	2.0	(0.0–24.0)	
June	1.0	(0.0–37.0)	

^a^Kruskal-Wallis test was used

^*^Statistically significant at < 0.05 level

#### Whole ALOS

For patients who did not undergo surgery while admitted (*n* = 700), the pooled whole ALOS over all included months and departments was 10 and ranged from 0 to 42 days.

From January to June, admission rates in non-operative patients were 18.3%, 22.1%, 18.9%, 12.4%, 14.2%, and 14.1%, respectively. Across the surgical units, 257 patients (20.0%) were admitted in Gynecology department, 308 patients (23.9%) in head and neck, 312 patients (24.2%) in GIT, 156 patients (12.1%) in Urology, 149 patients (11.6%) in Orthopedic, and 105 patients (8.2%) in Chest department.

A statistically significant difference in the whole ALOS was noticed between the 6 months (*p* value < 0.001). on pairwise adjusted comparisons, the Significantly different months were January vs. June (adjusted *p* value = 0.047), February vs. June (adjusted *p* value = 0.002), February vs. April (adjusted *p* value = 0.015), February vs. May (adjusted *p* value = 0.039). An apparent significance was noticed between ALOS in the surgical departments (*p* value = 0.046) but no statistical significance was found after applying pairwise adjusted comparisons.

### COVID-19 infection in relation to ALOS

Out of 1287 included patients from January to June, 28 positive COVID-19 patients (2.2%) were found in the 6 surgical departments from January to June. No significant difference was found as regards COVID-19 infection rates between the 6 months.

Statistical analysis did not show a significant link between COVID-19 infection rate and preoperative or whole ALOS duration neither collectively nor per month.

**Fig. 4:**
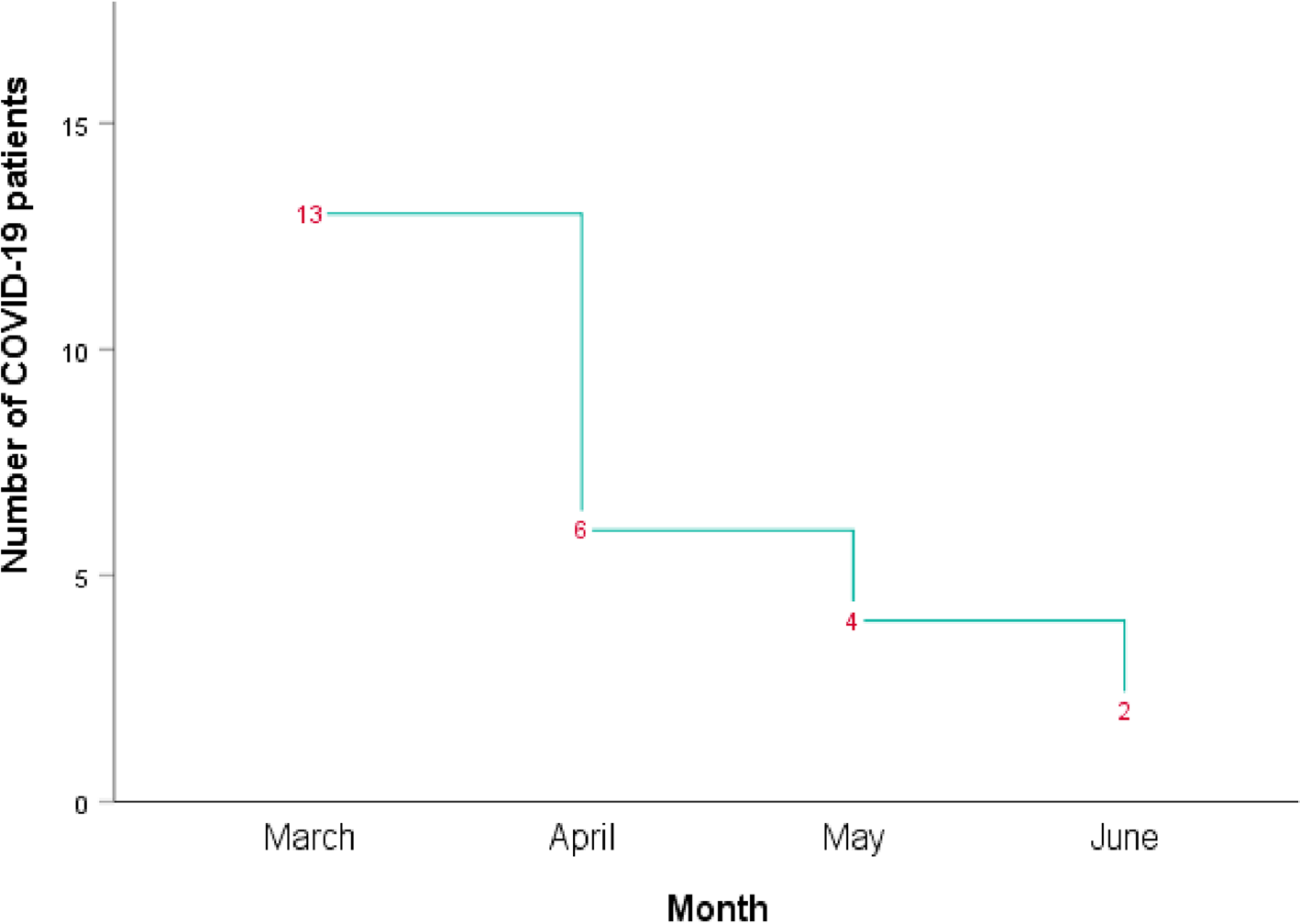
Linear trend of COVID-19 infection among admitted patients from March to June at NCI (*n* = 767), *p* value = 0.009

The separate analysis of the period from March to June (the 3rd wave of COVID-19 in Egypt) showed that 25 positive COVID-19 patients out of 767 admitted patients (3.3%) were encountered. Despite the non-significant difference in COVID-19 infection rates across the 4 months (*p* value = 0.076), a highly significant linear by linear association/trend was encountered (*p* value = 0.009) (Fig. 4).

Median duration between the negative and positive swabs (in COVID-19-confirmed patients) was 6 days and ranged from 2 to 34 days. Less COVID-19 positive cases (*n* = 8) were found when re-swabbing was done within 5 days than when it was done after a more prolonged period (*n* = 17).

From January to June, 5/587 operative patients (0.9%) were positive for COVID-19. Whole ALOS in the positive COVID-19 cases was 11 days versus 10 days for negative cases (*p* value = 0.485), while preoperative ALOS in the positive COVID-19 patients was 6 days versus 2 days for negative cases (*p* value = 0.337).

## Discussion

With the dual challenge of shrinking finances and growing population needs, the health sector finds itself under pressure. Reducing ALOS is an effective way of containing this growing demand for beds as well as releasing capacity in the health system. However, there is often significant variation in ALOS between and within hospitals, suggesting the need for improvements in internal processes, tracking systems, and the development of alternative services [7].

HAI also imposes costs on both patients and society in the form of increased resources, loss of time and effort, in addition to psychological and emotional suffering. Relation between LOS and HAI is interrelated. In many cases, the onset of HAI extends ALOS, and in other cases, the increased ALOS may increase the probability of HAI [8].

Since the COVID-19 pandemic in 2020, continuous risk assessment and management strategies have been generated and applied for early diagnosis, containing and controlling infection [9, 10].

The risk of COVID-19 infection is associated with epidemiological factors, host status, immunity, age, overall health, etc. [11, 12]. It is critical to protect susceptible populations by eliminating the transmission risks and benefit from hazard control practices [13] so patients having cancer and undergoing surgery were targeted in this study as a high-risk group that requires special care for prevention and control of such infection.

Out of all included patients from January to June, only 2.2% of patients were positive for COVID-19 infection. This was lower than a similar study on cancer patients documented a 7.8% infection rate [14].

The whole ALOS was 10 days in the current study. This was similar to what is documented by the previous study [15] with ALOS of 10.3 days and comparable to what is in another study [16], with 9.1 days.

In this study, ALOS reached a maximum of 42 days in some patients. This number lies in the previously described range by a systematic review of 52 published articles, with a maximum ALOS varied from less than a week to nearly 2 months [17].

In the current study, there was a statistically significant difference in preoperative ALOS across the 6 months (*p* value < 0.001) with significantly lower ALOS in the later months (April, May, and June) than in the former months (January–February, and March) which is linked to the continuous and effective risk management and monitoring during this period.

Despite the less common use of preoperative ALOS as an indicator of efficient risk management than the postoperative and whole ALOS, it is considered a better indicator in this study. This is due to the presence of many confounding factors that may interact and prolog the whole ALOS (e.g., different stages of disease requiring different treatment strategies, presence of medical or nonmedical conditions leading to postponed surgery), as well as the post-operative ALOS (e.g., postoperative complications, in compliance to treatment or medical instructions).

No significant difference was found among the 6 surgical departments (*p* value = 0.423) which could be explained by the consistent application of risk management measures across all departments.

Regarding COVID-19 infection, only 28 out of the 1287 patients became positive after admission. As expected, a small number of COVID-19 patients (*n* = 3) were infected during (January and February, the inter-wave duration) and 25 were infected (from March to June, the 3rd wave duration).

Combined analysis of COVID-19 infection rates across the 6 months pooling the inter-wave duration together with the duration of the 3rd wave might have masked a significant change in COVID-19 infection across the 6 months, so a separate analysis of the 3rd wave duration (from March to June) was done and it revealed a statistically significant decreasing trend of infection (*p* value = 0.009). This decreasing trend was opposite to the rising curve of infection by that time. A finding can be explained by the decreasing admission rates during the same duration as well as endorsing the efficiency of risk management and infection control measures as well as the lowered ALOS during this period.

Failure of spotting a significant link between COVID-19 infection rate and ALOS duration is explained by the small number of infected cases that could have hindered observing a possible significant relation.

Average duration between negative and positive swabs was 6 days with a lower number of positive COVID-19 patients when re-swabbing was done within 5 days than when done after 5 days, so shortening of the re-swabbing period could lower the chance of COVID infection through early identification and isolation of positive patients.

## Conclusion

ALOS (from January to June 2021) had significantly improved with commitment to risk management strategies. Targeting and minimizing ALOS was associated with a significant trend of decrease in COVID-19 infection (from March to June). Sticking to the 5-day swabbing duration could be helpful in minimizing the chance of COVID-19 infection through early detection and management of positive cases.

### Recommendations

In addition to the general measures directed to prevent and control COVID-19 infection, efforts targeting the average length of hospital stay as a hospital-based preventive measure in healthcare facilities - especially when dealing with cancer and low immunity patients -should be also encouraged. Continuously updated risk assessment and management strategies should be implemented and monitored. Further larger-scale studies with the evaluation of the COVID-19 infection during and before waves should be performed to establish a clear cause-effect relationship with ALOS and to ensure better generalizability of findings.

### Study strengths and limitations

Availability of a large sample size added more power to the study and better generalizability of results. High quality of tracking and monitoring data prevented missing data and provided a robust analysis. A small number of COVID-19 patients in the 2 months before the 3rd wave might have masked a significant change in COVID-19 infection across the whole study period from January to June.

## Data Availability

ALOS Data was regularly collected and analyzed by the Cancer Epidemiology and Biostatistics Department. COVID-19 infection data were collected by the IC unit, managed, and analyzed by the Cancer Epidemiology and Biostatistics Department.

## Abbreviations

ALOS: Average length of stay
HAI: Health-acquired infections
NCI: National Cancer Institute
IC: Infection control
COVID-19: Coronavirus disease of 2019
